# Profiling early adopters of ‘iSupport-Portugal’: a country-specific version of a worldwide adapted digital support program for informal caregivers of people with dementia

**DOI:** 10.3389/fpsyg.2024.1359695

**Published:** 2024-05-01

**Authors:** Soraia Teles, Sara Alves, Oscar Ribeiro, Alberto Freitas, Ana Ferreira, Constança Paúl

**Affiliations:** ^1^Department of Behavioral Sciences, School of Medicine and Biomedical Sciences, University of Porto (ICBAS-UP), Porto, Portugal; ^2^Center for Health Technology and Services Research (CINTESIS), Porto, Portugal; ^3^Center of Research, Diagnosis, Training and Care of Dementia (CIDIFAD), SCMRA, Riba D’Ave, Portugal; ^4^Department of Education and Psychology, University of Aveiro, Aveiro, Portugal; ^5^Faculty of Medicine, University of Porto (FMUP), Porto, Portugal

**Keywords:** caregivers, dementia, digital technologies, mental health, remote measurement

## Abstract

**Introduction:**

Informal caregivers are the backbone of dementia care. iSupport is a World Health Organization digital support program for caregivers of people with dementia (PwD) that has been culturally adapted in several countries. iSupport was previously assessed for its feasibility in Portugal, and this country-specific version is now being utilized as a remote measurement tool (RMT). It constitutes the first internationally developed iSupport platform that is technically and scientifically enhanced to collect data on sociodemographic, clinical, and psychosocial variables of dementia care dyads. This paper characterizes the early adopters of iSupport-Portugal and discusses its exploration as a RMT.

**Methods:**

Cross-sectional data were collected between February and July 2023 from users registering on isupport-portugal.pt. To characterize caregivers and PwD, eligibility was limited to unpaid caregivers assisting community dwelling PwD (*n* = 173). Data were collected through self-administered instruments in users’ accounts. Caregivers completed psychosocial measures on burden, anxiety, depression, quality of life, desire to institutionalize and usage of community services. Textual data on caregivers’ needs underwent content analysis.

**Results:**

Among the early adopters of iSupport-Portugal (*n* = 365), 52.3% were informal caregivers, while 44.7% were health/social care professionals or others. Most caregivers were female (82.7%), middle-aged (M 51.7 years), highly educated (M 15.3 years) and supporting a parent (70.5%). Caregivers cared for a median of 24 h/week and 60.8% lived with the PwD. Neuropsychiatric symptoms were reported for 94.1% of PwD, who scored as moderately dependent (Barthel Index: M 14.0). Significant burden was reported by 88.4% of caregivers (≥21 on ZBI-22). Among caregivers scoring borderline or abnormal (≥8 on HADS) for anxiety, depression, or both (75.5%), 30.8% sought mental health counseling. Caregivers supporting a PwD not using community services scored higher on anxiety (*p* = 0.003), and depression (*p* = 0.009). Text data revealed unmet practical, emotional, and informational needs.

**Discussion:**

iSupport-Portugal has garnered fair initial interest from caregivers, particularly from those who are children, highly educated, and employed. Early adopters exhibited significant psychological distress, and both practical and emotional needs, which contrast with limited use of support services for themselves and the PwD. iSupport-Portugal shows promise for descriptive research on care dyads, particularly among newer generations of caregivers.

## 1 Introduction

Dementia represents a significant global public health challenge, affecting approximately 55 million individuals worldwide (World Health Organization, 2021). In 2019, Portugal stood as the fourth-ranked country among OECD nations in terms of dementia prevalence, with an estimated rate of 21 cases per 1,000 inhabitants (OECD, 2019).

As dementia stands as the primary cause of dependence among older adults (Sousa et al., 2010), people with dementia (PwD) often require consistent care. A significant 84% of PwD worldwide live at home, where they rely on assistance primarily provided by family members, neighbors, or friends (Wimo et al., 2018). These supporters, commonly referred to as informal caregivers, shoulder the responsibility of unpaid and continuous assistance in basic or instrumental activities of daily living and/or in organizing care delivery by others. Informal caregivers worldwide serve as the linchpin of the care and support system, playing a pivotal role in enabling individuals to age in their own homes. Nonetheless, informal caregivers of PwD are at greater risk of experiencing depression and anxiety disorders, as well as hypertension, digestive, and breathing problems when compared both to the general population and to caregivers of people living with other chronic diseases (WHO, 2015; Gilhooly et al., 2016). These health issues often coexist with strained relationships, social isolation, and financial hardships. Dementia is linked to the necessity for particularly intensive and multifaceted care and its progressive nature. The still limited availability and uncertainties over recent disease-modifying treatments (NHS, 2024), and the complex psychological and behavioral symptoms are all instances of the distinctive challenges faced by dementia caregivers (Schulz et al., 2020).

**Figure 1:**
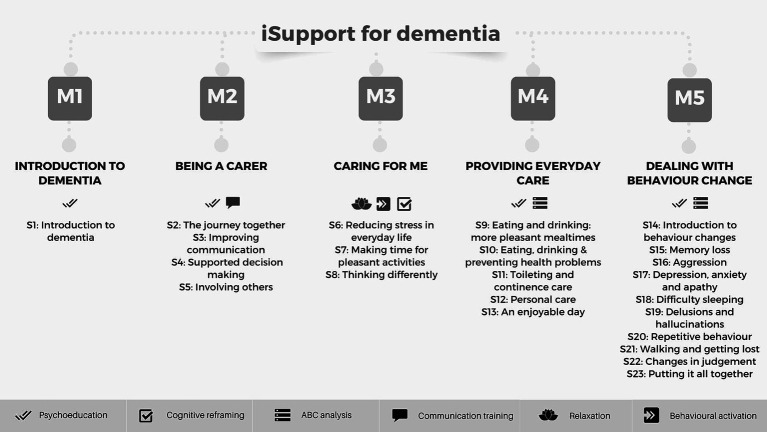
iSupport modules, lessons, and psychological techniques. Lessons names vary slightly in the European-Portuguese version.

Reflective of this evidence, the World Health Organization (WHO) Global Action Plan on the Public Health Response to Dementia has established the goal of having 75% of countries offering accessible support and training programs by 2025 to mitigate the adverse consequences of caregiving (WHO, 2017). As part of this plan, WHO has developed “iSupport for dementia,” an eHealth program for caregivers of PwD (Pot et al., 2019). Additionally, iSupport was introduced in the form of a hardcopy manual to accommodate individuals facing challenges such as limited internet access or insufficient digital skills. The philosophy underlying iSupport aligns with Kitwood’s model, emphasizing the centrality of personhood for individuals diagnosed with dementia (Kitwood, 2017). Care is thought of as interaction in accordance with the needs, abilities, and personality of each individual (Kitwood, 2017). Across the 23 lessons and 5 modules comprising the iSupport program, problem-solving and cognitive-behavioral therapy techniques are employed. These include psychoeducation, behavioral activation, cognitive reframing, relaxation, communication training, and antecedent-behavior-consequence (ABC) analysis (see Figure 1).

The digital program was initially designed to be freely accessible and self-guided. Caregivers can navigate the content independently, while selecting the lessons that best suit their needs and establishing personalized schedules for accessing the program. Informal caregivers frequently face substantial challenges in accessing conventional interventions. This is attributed to factors such as limited operating hours, difficulties coordinating with employment or full-time care responsibilities, and transportation issues (Pot et al., 2015; Teles et al., 2021). An online and self-guided program offers full flexibility regarding the intervention schedule, enabling individuals to progress at their own pace. Self-guided online interventions may improve the accessibility of psychosocial support. Additionally, they may contribute to reducing the costs associated with assisting an expanding number of PwD and their caregivers (Blom et al., 2015). To elevate engagement and user experience in iSupport, the program incorporates personalization features. Furthermore, iSupport integrates caregiving scenarios that replicate real-life situations, linking these scenarios to interactive exercises for skills training. The primary goal is to assist caregivers in internalizing the underlying theory presented in the lessons.

**Figure 2:**
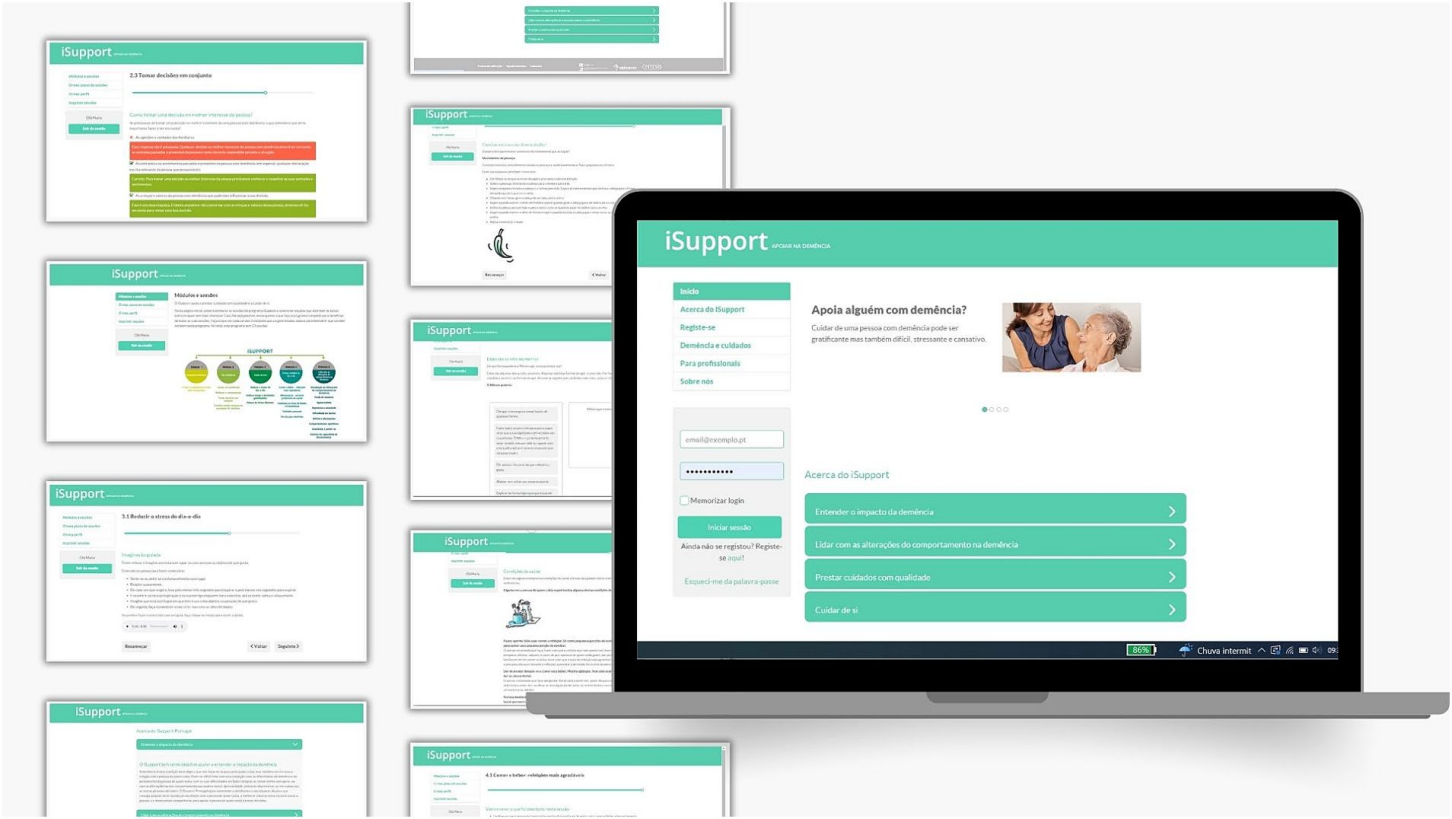
iSupport-Portugal screens (at isupport-portugal.pt).

iSupport was originally crafted as a ‘generic version’ presented in English and featuring examples and scenarios from various cultures. Therefore, cultural adaptation to each implementation setting is necessary. This digital program aimed at improving the mental health of informal caregivers has been or is being adapted in over 40 countries. iSupport-Portugal (see Figure 2) stands as one of the pioneering culturally adapted versions (Teles et al., 2020) which was studied for its usability (Teles et al., 2021) and feasibility (Teles et al., 2022), yielding promising results. Other country-specific versions of iSupport have published results regarding their cultural adaptation, including for Australia (and Chinese-Australian caregivers (Xiau, 2020; Xiao et al., 2022)), Brazil (Oliveira et al., 2020), India (Baruah et al., 2021), Switzerland (Fiordelli and Albanese, 2020), Indonesia (Turana et al., 2023), Spain (Molinari-Ulate et al., 2023), Greece (Efthymiou et al., 2022) and Japan (Yamashita et al., 2022).

iSupport-Portugal is currently undergoing exploration beyond its initial intervention purpose. It is evolving into a research-intervention platform with the potential to remotely assess the sociodemographic, health, and psychosocial aspects of caregivers and PwD, nationwide and longitudinally. Web platforms and mobile apps have seen growing exploration as remote measurement tools or technologies (RMT). RMT provide alternatives to traditional and frequently cumbersome assessment methods by facilitating real-time and longitudinal monitoring of health variables and behaviors in a cost-effective and non-intrusive manner (Simblett et al., 2018). iSupport-Portugal is the first internationally developed iSupport platform that is technically and scientifically enhanced to collect and export data on sociodemographic, clinical, and psychosocial variables of dementia care dyads. This includes response data to surveys and scales, replies to interactive exercises, and paradata, i.e., actions on the interface, such as pages visited, and time spent on pages. The use of iSupport for collecting data for descriptive and predictive research on dementia care dyads is currently under exploration.

Many countries, including Portugal, lack national data on informal caregivers of PwD, including their number, characteristics, and the care they provide (World Health Organization, 2021). While a recent national survey to informal caregivers indicated that dementia is the primary condition among care recipients (33%), the psychosocial profile of these caregivers remains undescribed (Movimento cuidar dos cuidadores informais, 2021). Regional or national projects have depicted caregivers of PwD as predominantly female (Gonçalves-Pereira et al., 2019; Paúl et al., 2019), spouses or children (Gonçalves-Pereira et al., 2019; Paúl et al., 2019), with lower levels of education (Gonçalves-Pereira et al., 2019; Paúl et al., 2019), and mostly unemployed (Paúl et al., 2019). However, due to availability, caregivers in intervention programs [e.g., Paúl et al. (2019)] may be more likely to be unemployed, providing full-time care, and have lower levels of education, as more educated caregivers are less prone to leave their jobs for full-time care (Flinn, 2018). A recent cohort study in Portugal observed a higher-than-expected percentage of participants with secondary or higher education (Gonçalves-Pereira et al., 2019). This observation may indicate a changing profile of informal caregivers. Current international reports have highlighted the emerging generation of family caregivers, who tend to be more schooled, employed, and the only children of the care recipient (Flinn, 2018; National Alliance for Caregiving, AARP, 2020). As the profile of caregivers continues to evolve, tools for collecting data on their characteristics and needs can be valuable for planning the organization of healthcare services.

This paper aims to provide a comprehensive characterization of the sociodemographic, clinical, and psychosocial profiles of informal caregivers and PwD, along with their utilization of community resources and caregivers’ needs, among users of iSupport-Portugal. This digital platform and support program was utilized as a RMT to collect nationwide data on dementia care dyads at a distance.

## 2 Materials and methods

### 2.1 Preliminary measures

**Figure 3:**
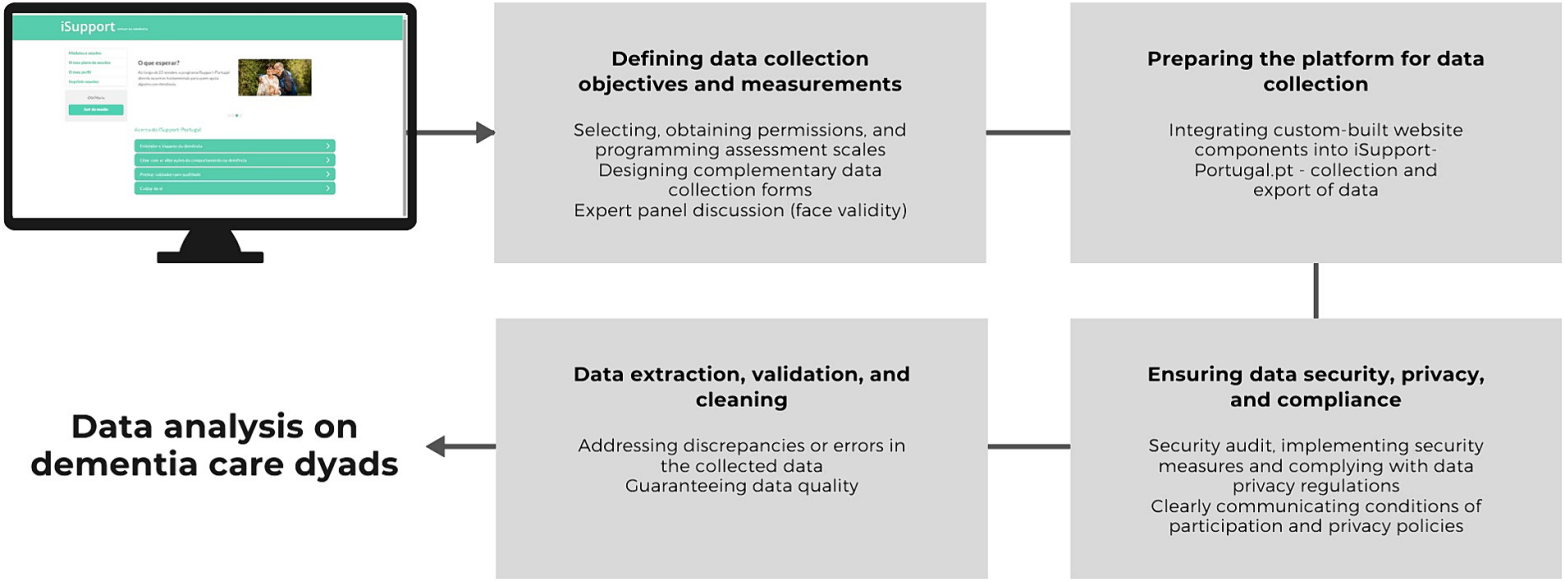
Preliminary measures to exploit iSupport-Portugal as a RMT for collecting data on dementia care dyads.

To exploit iSupport-Portugal as a RMT for collecting data on dementia care dyads, a series of preparatory steps were undertaken before the data analysis phase (see Figure 3).

First, these encompassed clearly defining data collection objectives and measures, as well as selecting, obtaining permissions for use, and programming assessment scales in accordance with licensing requirements. Simultaneously, this step involved designing complementary data collection forms, utilizing suitable form fields and validation to improve data accuracy. Measures were defined based on a literature review and previous iSupport-Portugal research. A mixed-methods pilot RCT, which included most of the measures utilized for the present research, provided insights into the adequacy of these measures (Teles et al., 2022). Furthermore, an expert panel discussion focused on the utility of embedded exercises within iSupport as information sources. These include, the open-text exercise providing data on caregivers’ needs, as reported in section 3.4.

Second, the platform was prepared for data collection. This required integrating custom-built website components into iSupport-Portugal.pt. to facilitate the collection and export of data, as well as background analytics on survey use. Furthermore, proper, and secure data storage and backup mechanisms were put in place.

Third, steps were taken to ensure data security, privacy, and compliance, given the objective of collecting health and well-being data on dementia care dyads. A thorough analysis of the platform’s security to identify vulnerabilities was performed and measures were implemented to enhance data security. These included encryption for data transmission, secure storage practices, and access controls to prevent unauthorized access. Compliance with data privacy regulations such as General Data Protection Regulation (GDPR) was ensured. The necessary consents from participants were obtained while guaranteeing a clear communication of participation conditions and privacy policies. To warrant compliance with data protection regulations and the appropriateness of informed consents, support was sought from the Data Protection office and the digital services of the University of Porto.

Fourth, data extraction, validation, and cleaning were performed to address any discrepancies or errors in the collected data. While data cleaning is a fundamental process for any dataset, doing so for online platforms demands considerable effort and time due to the large quantity and diversity of data.

All these systematic steps ensure the integrity, security, and ethical handling of the data collected through iSupport-Portugal. They provide the basis for robust and reliable analysis of data from dementia care dyads.

### 2.2 Design

Observational study, with cross-sectional primary data collected at registration to the online platform isupport-portugal.pt.

### 2.3 Participants and recruitment

All individuals who completed registrations on isupport-portugal.pt. between February and July 2023 were included in the analysis to characterize the user base of this platform. To characterize the sociodemographic, clinical, and psychosocial profile of caregivers and care recipients, eligibility was limited to i. adults (18 years and older), ii. resident in Portugal, iii. providing unpaid support, iv. to a person diagnosed with dementia, v. living in the community (i.e., not in permanent institutional care). Registered users discovered the platform through various dissemination channels. These include the websites of the program organizers and partners, media press articles, and recommendations from professionals.

### 2.4 Variables and measures

The study data were collected exclusively online through fill-in forms hosted at isupport-portugal.pt. To explore iSupport as a RMT, a diagnosis module was incorporated into the program, making it a unique addition to iSupport-Portugal compared to other international versions. The diagnosis module serves as the baseline assessment protocol of sociodemographic, clinical, and psychosocial variables pertaining to the caregivers and PwD. Therefore, it includes the selected measures outlined in sections 2.4.1 and 2.4.2. This module precedes the five intervention modules comprising iSupport. It is available and can be self-completed by the participants after giving their consent to participate in research, all through their user account. Once caregivers complete the registration process and consent to their participation in research, they are prompted to navigate and fill in questions within the diagnosis module. All data about PwD were collected through the caregivers’ report. The instruments administered to the study participants are described in Table 1.

**Table 1:** Instruments administered to study participants.

**Instrument**	**Description**
**Sociodemographic characteristics of IC and PwD**	
Sociodemographic questionnaire	All registered users: age, gender, years of formal education, region of residence (NUTS II) IC and PwD: marital status, IC-PwD relationship/kinship IC only: employment status, number of children and number of cohabiting children
**Care context**	
Care context questionnaire	Caregiving duration, hours spent caring per week, access to regular support for caregiving and (if so) type of support (unpaid, paid and specialized, paid but unspecialized), cohabitation with the PwD
**Use of support services for IC and PwD**	
Service utilization items	IC: current use of psychoeducational, support or mutual aid groups, mental health counseling, or other PwD: current use of home care services, home health services, day or night centers, respite services, cognitive or occupational therapy, or other
**Clinical profile of PwD**	
PwD clinical profile questionnaire	Type of dementia, time since diagnosis of dementia, level of dependency perceived by the caregiver.
Barthel index	Mahoney and Barthel (1965) / Portuguese version by Araújo et al. (2007) A 10-item instrument that assesses functional independence; items are scored from 0 to a maximum of 3, with total scores ranging from 0 to 20. Higher scores indicate greater independence. Cut-offs for dependence levels are total dependence (0–8 points), severe dependence (9–12 points), moderate dependence (13–19 points) and independent (20 points).
Neuropsychiatric inventory questionnaire (NPI-Q)	Cummings et al. (1994) / Portuguese version by Espirito-Santo et al. (2010) Assesses the presence or absence of 12 neuropsychiatric symptom domains: delusions, hallucinations, agitation/aggression, dysphoria/depression, anxiety, euphoria/elation, apathy/indifference, disinhibition, irritability/lability, aberrant motor behaviors, nighttime behavioral disturbances, and appetite/eating disturbances. The severity of reported symptoms in the past month is rated as mild, moderate, or severe, with the total NPI-Q severity score ranging from 0 to 36. Caregiver distress for each symptom reported is rated on a 6-point scale, with total NPI-Q distress scores ranging from 0 to 60.
**Psychosocial profile of IC**	
Zarit Burden interview (ZBI-22)	Zarit et al. (1980) / Portuguese version by MAPI Research Trust (2014) 22-item instrument assessing caregiver perceived burden; items are scored on a 5-point scale and the total ZBI score ranges from 0 to 88 points. Higher scores indicate greater burden.
Hospital anxiety and depression scale (HADS)	Zigmond and Snaith (1983) / Portuguese version by Pais-Ribeiro et al. (2007) A 14-item instrument that assesses symptoms of anxiety and depression in two subscales, each with 7 items scored on a 4-point scale; total scores per subscale range from 0 to 21. Higher scores indicate more severe symptoms of anxiety or depression.
WHOQOL-BREF	World Health Organization (1996) / Portuguese version by Vaz Serra et al. (2006) A 26-item instrument covering four domains of quality of life: physical, psychological, social relationships and environment, as well as items relating to overall quality of life. Each item is rated on a 5-point scale. Higher total scores indicate higher quality of life.
Positive aspects of caregiving (PAC)	Tarlow et al. (2004) / Portuguese version by Gonçalves-Pereira et al. (2010) An 11-item instrument assessing positive feelings resulting from caregiving; items are scored on a 5-point scale, with total scores ranging from 11 to 55. Higher scores represent more positive perceptions of caregiving.
Desire to institutionalize scale (DIS)	Morycz (1985) / Portuguese version by Teles et al. (2023) A 6-item scale assessing different stages of contemplating institutionalization; dichotomous response option (‘yes’ = 1 point; ‘no’ = 0 points) with an overall desire to institutionalize score ranging from 0 to 6 points. Higher scores indicate a greater willingness to institutionalize the PwD.
**Unmet needs of IC**	
Key caregiver needs	Session 2.4 of iSupport; non-mandatory exercise consisting of identifying the three main needs of caregivers (text entry/data).

IC, informal caregiver; PwD, person with dementia.

#### 2.4.1 Data on informal caregivers

Informal caregivers provided sociodemographic information about themselves and details about the context of care. Use of services for caregivers was assessed, including psychoeducational, support or mutual aid groups, mental health counseling, or others. In addition, participants completed several psychosocial measures: i. the Zarit Burden Interview (ZBI-22) (Zarit et al., 1980; MAPI Research Trust, 2014); ii. the Hospital Anxiety and Depression Scale (HADS) (Zigmond and Snaith, 1983; Pais-Ribeiro et al., 2007); iii. The WHOQOL-BREF (World Health Organization, 1996; Vaz Serra et al., 2006); iv. the PAC (Tarlow et al., 2004; Gonçalves-Pereira et al., 2010); and v. the European-Portuguese version of the Desire to Institutionalize Scale (Morycz, 1985; Teles et al., 2023).

#### 2.4.2 Data on persons with dementia

Caregivers provided sociodemographic and clinical information about the PwD in their care and reported on the use of services, including home care services, home health services, day, or night centers, cognitive or occupational therapy, or other. The Barthel Index (Mahoney and Barthel, 1965; Araújo et al., 2007) and the Neuropsychiatric Inventory Questionnaire (NPI-Q) (Cummings et al., 1994; Espirito-Santo et al., 2010) were also completed by caregivers.

### 2.5 Data analysis

Descriptive statistics were calculated, utilizing absolute and relative frequencies, as well as measures of central tendency and dispersion, where appropriate. For interpretability, raw scores on each WHOQOL-BREF domain were transformed into a scale between 0 and 100 according to the scoring guidelines (World Health Organization, 1996). Relationships between theoretically relevant variables and group differences were examined using parametric or non-parametric tests (Spearman’s rank, Pearson’s, or Kendall’s tau-b correlation; chi-squared test for independence; independent samples *t*-test, Kruskall-Wallis, or Mann–Whitney *U* test), according to the conditions of applicability. All *p*-values are two-tailed with a significance level of 0.05. The Statistical Package for the Social Sciences/IMB SPSS Statistics version 27 (IBM Corp, 2020) (RRID:SCR_002865) was used for analysis.

The text data on the key needs of caregivers were subjected to thematic content analysis using NVivo software, version 11. The content was coded in categories defined in an inductive/data-driven approach. Results are presented as absolute frequencies for references coded by category. Text excerpts (translated into English) are used to illustrate content within categories.

### 2.6 Ethics and data protection

Upon registration at isupport-portugal.pt. to access the online program, all users consented to the use of their basic sociodemographic data entered in the registration form and their navigation data for research purposes. Additionally, users who self-identified as unpaid caregivers of a PwD were fully informed and invited to participate in the research by completing post-registration questionnaires. Informed consent was obtained online through the user’s personal account at isupport-portugal.pt. The refusal to participate in the study did not impede the use of the program in any way. A pseudonymization process was implemented. This study was approved by the Ethics Committee for Health of the Faculty of Medicine of the University of Porto (ref: 76/CEFMUP/2022). An assessment of data protection issues for isupport-portugal.pt. was carried out by the Data Protection Officer of the University of Porto.

## 3 Results

### 3.1 Quantitative analysis

#### 3.1.1 Registered users on iSupport-Portugal

Between February and July 2023, a total of 449 users registered on isupport-portugal.pt., 365 of whom completed the basic sociodemographic form presented at registration. Of these, 191 (52.3%) registered as informal caregivers of a PwD, 11 as paid caregivers (3.0%) and 163 (44.7%) as health/social support professionals or others.

#### 3.1.2 Sociodemographic characteristics and care context

Among the eligible caregivers (*N* = 173), not all completed the baseline measures in full. For sociodemographic variables collected through the registration form (e.g., age), there are significantly more cases compared to other variables collected through post-registration questionnaires (see Table 2).

**Table 2:** Summary of sociodemographic variables for caregivers and PwD and context of care variables.

**Variables**	***N***	**Descriptive statistics**
**Informal caregivers**		
Age (years), *M* (SD)	173	51.7 (13.0)
Gender, Female, *n* (%)	173	144 (83.2)
Years of formal education, *M* (SD)	171	15.3 (4.4)
Marital status, Partnered ^a^, *n* (%)	97	61 (62.9)
Employment status, Employed, *n* (%)	98	63 (64.3)
Relationship with the care recipient	173	
Offspring, *n* (%) ^b^		132 (76.3)
Spouses, *n* (%)		23 (13.3)
Other, *n* (%)		18 (10.4)
Children, Yes		
Among all carers, *n* (%)	96	63 (65.6)
Among offspring caregivers, *n* (%)	72	43 (59.7)
Among spousal caregivers, *n* (%)	16	14 (87.5)
Cohabiting children, Yes		
Among all caregivers with children, *n* (%)	63	41 (65.1)
Among offspring caregivers with children, *n* (%)	43	32 (74.4)
Among spousal caregivers with children, *n* (%)	14	4 (28.6)
**Person with dementia**		
Age (years), *M* (SD)	173	78.8 (8.5)
Gender, Female, *n* (%)	173	109 (63.0)
Years of formal education, Mdn (IQR)	97	4 (5)
Marital status, Partnered^†^, *n* (%)	97	57 (58.8)
**Informal care context factors**		
Caregiving duration (months), Mdn (IQR)	94	33 (58)
Hours caring (per week), Mdn (IQR)	96	24 (45.8)
Support for caregiving, Yes, *n* (%)	97	67 (69.1)
Support for caregiving, type of support	67	
Unpaid, *n* (%)		37 (55.2)
Paid, specialized, *n* (%)		11 (16.4)
Paid, non-specialized, *n* (%)		19 (28.4)
Cohabitation with the PwD, Yes, *n* (%)	97	59 (60.8)

N/n, number of participants; M, mean; Mdn, median; SD, standard deviation; IQR, interquartile range.

^a^ Includes those who were married or in a de facto union; ^b^ Includes children and grandchildren.

Most caregivers are female (*n* = 143, 82.7%), middle-aged (*n* = 173, M 51.7, SD 13.0, range: 20–89 years) and were caring for a parent (*n* = 122, 70.5%). On average, caregivers were highly educated (*n* = 171, M 15.3 years of education, SD 4.4, range: 3–25) and most were employed (*n* = 63, 64.3%). Caregiver education negatively correlates with the hours spent caring for the PwD (*n* = 96, r_s_ = −0.295, *p* = 0.004).

Care recipients are predominantly female (*n* = 109, 63.0%) and had a mean age of 78.8 years (*n* = 173, SD 8.5). The age range of PwD at the time of data collection (45–96 years) suggests a representation of young onset dementia cases.

More than half of the caregivers lived with the PwD (*n* = 59, 60.8%). Most had been providing care for two or more years (*n* = 94, 60.6%; Mdn 33 months, IQR 58) and were spending 20 h or more per week providing care (*n* = 96, 61.5%; Mdn 24 h, IQR 45.8). While most caregivers were supported in their caring responsibilities (*n* = 67, 69.1%), more than half received support from other unpaid caregivers (55.2%).

#### 3.1.3 Clinical profile of PwD and service use

According to caregivers, most care recipients had been diagnosed with Alzheimer’s disease (*n* = 46, 46.9%). The median time since diagnosis was 41 months (*n* = 97, IQR 59.5). According to the Barthel Index cut-off scores, almost half of the PwD (*n* = 35, 44.9%) would be classified as moderately dependent (*n* = 78, Mdn 14, IQR 13). However, the sample is diverse, with PwD distributed across all levels of dependence (Barthel Index range: 0 to 20 points). There is a strong negative correlation between the perceived level of dependence of the PwD and the total score on the Barthel Index (*τ*_b_ = 0.600, *p* < 0.001). At least one neuropsychiatric symptom was reported by 94.1% (*n* = 74) of caregivers on the NPI-Q, with a median of 5 symptoms (IQR 4, range: 0–12), and a median severity score of 10 (IQR 9.0). The most reported neuropsychiatric symptoms were apathy (*n* = 63, 80.8%), appetite changes (*n* = 42, 53.8%) and depression (*n* = 41, 52.6%), while euphoria was the least reported (*n* = 10, 12.8%). The positive symptoms that scored higher on severity were apathy (*n* = 63, M 2.22, SD 0.66), motor disturbances (*n* = 33, M 2.06, SD 0.75), delusions (*n* = 27, M 2.04, SD 0.71) and agitation (*n* = 27, M 2.04, SD 0.65).

Almost half of the participants (*n* = 46, 48.4%) reported that the PwD did not use any of the services listed in Table 3. Home care services were the most used (*n* = 19, 20.0%).

**Table 3:** Summary of the PwD clinical profile variables and service use.

**Variable**	***N***	**Descriptive statistics**
Type of dementia	98	
Alzheimer’s disease, *n* (%)		46 (46.9)
Vascular dementia, *n* (%)		17 (17.3)
Frontotemporal dementia, *n* (%)		12 (12.2)
Dementia with Lewy bodies, *n* (%)		8 (8.2)
Other/unknown, *n* (%)		15 (15.3)
Time since diagnosis (months), Mdn (IQR)	97	41 (59.5)
Dependence level, perceived by the carer		
Mild, *n* (%)		16 (16.5)
Moderate, *n* (%)		32 (33.0)
Severe, *n* (%)		25 (25.8)
Total, *n* (%)		24 (24.7)
Functional independence (BI), Mdn (IQR)	78	14 (13)
Total dependence, *n* (%)		21 (26.9)
Severe dependence, *n* (%)		8 (10.3)
Moderate dependence, *n* (%)		35 (44.9)
Independent, *n* (%)		14 (17.9)
Neuropsychiatric symptoms (NPI-Q)	78	
Number of symptoms (NPI-Q), Mdn (IQR)		5 (4.0)
Severity (NPI-Q), Mdn (IQR)		10 (9.0)
Service use by the care recipient	95	
Home care services, uses, *n* (%) ^a^		19 (20.0)
Home health services, uses, *n* (%)		13 (13.7)
Day center, uses, *n* (%)		15 (15.8)
Night center, uses, *n* (%)		2 (2.1)
Cognitive or occupational therapy, uses, *n* (%)		11 (11.6)

*N*/*n*, number of participants; *M*, mean; Mdn, median; SD, standard deviation; IQR, interquartile range; BI, Barthel index; NPI-Q, neuropsychiatric inventory questionnaire.

^a^ includes homecare services with or without support for personal care (e.g., personal hygiene).

**Table 4:** Summary of psychosocial variables for caregivers.

**Variable**	***N***	**Descriptive statistics**
Perceived burden (ZBI-22), *M* (SD)	95	36.0 (12.9)
Caregiver distress, neuropsychiatric symptoms (NPI-Q), Mdn (IQR)	73	11.0 (13.0)
Anxiety symptoms (HADS-A), *M* (SD)	85	10.0 (4.2)
Depression symptoms (HADS-D), *M* (SD)	85	7.5 (4.2)
Quality of life (WHOQOL-BREF)	82	
General, *M* (SD)		6.8 (1.6)
Physical, *M* (SD)		25.5 (5.4)
Psychological, *M* (SD)		21.4 (4.0)
Social relationships, *M* (SD)		9.7 (2.5)
Environment, *M* (SD)		28.2 (5.7)
Positive aspects of caregiving (PAC), *M* (SD)	89	34.2 (10.0)
Desire to institutionalize the PwD (DIS-PT), Mdn (IQR)	88	2 (3)
Service use by the caregiver, uses, *n* (%)	95	
Mental health consultations		25 (26.3)
Psychoeducational, support or mutual aid groups, uses, *n* (%)		9 (9.5)
Holiday center or carer relief services, uses, *n* (%)		0

*N*/*n*, number of participants; *M*, mean; Mdn, median; SD, standard deviation; IQR, interquartile range; ZBI, Zarit Burden interview; HADS-A, hospital anxiety and depression scale (anxiety subscale); HADS-D, hospital anxiety and depression scale (depression subscale); NPI-Q, neuropsychiatric inventory questionnaire; PAC, scale positive aspects of caregiving positive; DIS-PT, desire to institutionalize scale, European-Portuguese version.

^a^ Reports on raw scores for each QoL domain.

#### 3.1.4 Psychosocial profile and service utilization of informal caregivers

##### 3.1.4.1 Burden of care, psychological distress, and service use

Significant levels of burden were reported by caregivers (*n* = 95, M 36.0, SD 12.9), with 88.4% (*n* = 84) scoring ≥21 on the ZBI-22 (Zarit et al., 1980). The distress caused by neuropsychiatric symptoms was on average higher for agitation/aggression (*n* = 27, M 2.81, SD 0.97), anxiety (*n* = 33, M 2.79, SD 0.86) and delusions (*n* = 27, M 2.78, SD 0.93). A moderate positive correlation between the ZBI-22 and the NPI-Q distress total scores (r_s_ = 0.411, *p* < 0.001) is observed.

According to the HADS cut-off scores, 44.7% (*n* = 38) of caregivers would classify as abnormal and 24.7% (*n* = 21) as borderline abnormal for anxiety. For depression, 20% (*n* = 17) would classify as abnormal and 27.1% (*n* = 23) as borderline.

The number of hours spent on caregiving is positively correlated with the level of burden (*n* = 94, r_s_ = 0.312, *p* = 0.002), anxiety (*n* = 85, r_s_ = 0.266, *p* = 0.014) and depression (*n* = 85, r_s_ = 0.336, *p* = 0.002). Likewise, the severity of neuropsychiatric symptoms is positively correlated with the level of burden (*n* = 76, r_s_ = 0.350, *p* = 0.002), anxiety (*n* = 77, r_s_ = 0.306, *p* = 0.007), and depression (*n* = 77, r_s_ = 0.304, *p* = 0.007). Caregivers who reported no support for caring responsibilities scored higher on burden (M 42.2, SD 12.4 vs. M 33.3 SD 12.1, *t* (93) = −3.280, *p* = 0.001), anxiety (M 11.6, SD 3.6 vs. M 9.3, SD 4.2, *t* (83) = −2.390, *p* = 0.019) and depression (M 8.9, SD 4.1 vs. M 6.9, SD 4.2, *t* (83) = −2.132, *p* = 0.036), than those receiving such support.

Of the caregivers who scored as borderline or abnormal for either anxiety, depression, or both (*n* = 65, 76.5%), only 30.8% (*n* = 20) were seeking mental health counseling. When asked about the use of support services, most participants reported using none (*n* = 54, 56.8%). None of the caregivers were using carer relieve services. Caregivers supporting PwD who were not using community services scored significantly higher on anxiety (M 11.3, SD 3.9 vs. M 8.6, SD 4.1, *t* (82) = 3.102, *p* = 0.003), and depression (M 8.7, SD 4.4 vs. M 6.3, SD 3.7, *t* (82) = 2.681, *p* = 0.009) than those who were.

##### 3.1.4.2 Quality of life

Transformed scores for the WHOQOL-BREF show that the social relationships domain is on average the lowest rated (*n* = 82, M 56.2, SD 21.1) compared to the physical (M 66.1, SD 19.1), psychological (M 64.0, SD 16.8), and environmental (M 63.1, SD 17.8) domains. The average transformed score for general QoL was 60.5 (SD 19.4). Of all three indicators of psychological distress (burden, anxiety, and depression), depression shows the strongest negative correlations with all QoL domains (*n* = 82, general r_p_ = −0.577, *p* < 0.001, physical r_p_ = −0.515, *p* < 0.001, psychological r_p_ = −0.744, *p* < 0.001, social r_p_ = −0.613, *p* < 0.001, and environmental r_p_ = −0.577, *p* < 0.001).

##### 3.1.4.3 Positive aspects of caregiving and desire to institutionalize

Despite high psychological distress, positive aspects of care were moderately rated (M 34.2 points on the PAC). Positive aspects were negatively correlated with the severity of neuropsychiatric symptoms (*n* = 77, r_s_ = −0.248, *p* = 0.030), and with the functional independence of the PwD (*n* = 78, r_s_ = −0.356, *p* = 0.001). Caregivers reported only mild desire to institutionalize (*n* = 88, Mdn 2, IQR 3). The willingness to institutionalize is positively correlated with the level of caregiver burden (*n* = 87, r_s_ = 0.415, *p* = 0.001).

### 3.2 Qualitative analysis of unmet needs among informal caregivers

**Figure 4:**
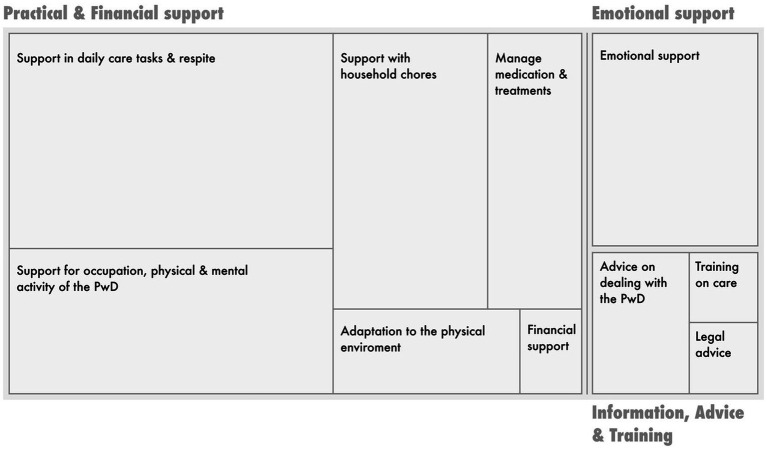
Visual representation of the number of items coded per coding category/node. Source: Nvivo 11, edited for improved visualization of category titles.

Through responses to open-text exercises in iSupport-Portugal, a sub-sample of caregivers (*n* = 20) reported unmet needs, coded as i. practical support (39 references), ii. emotional support (7 references), and iii. information, advice, and training support (5 references). In addition, the need for a better work-life balance has emerged (5 references) (see Figure 4).

The most reported need was for practical support in caring. Extracts within this category highlight caregivers’ needs for i. support with daily tasks, including bathing and other personal care tasks, and ii. for time out from caring: e.g. *“I would need at least one afternoon a week to myself”* (IC_414, daughter). The provision of occupational activities and increasing the physical/mental activity of the PwD also emerged as a concern: e.g. *“I would need someone to take my dad for a walk a couple of times a week or to accompany him in the activities he likes best when I’m at work”* (IC_781, daughter). Assistance with household tasks, including cleaning, meal preparation and grocery shopping was a common need. Financial support needs were the least expressed, although assistance with household chores or respite care most often depended on the family’s financial resources.

Emotional support needs expressed by caregivers included support both from other caregivers – *“I would need to talk to someone who is going through the same difficulties”* (IC_272, granddaughter) and from mental health professionals – *“I would need psychological help, it’s extremely difficult to live with a person in this [referring to dementia] situation. I understand and accept the illness, but at the same time I feel exhausted, tired and I’ve asked myself how much longer I can put up with it”* (IC_824, husband). The need for advice on how to deal and communicate with the PwD, how to provide practical care, and how to activate legal mechanisms such as power of attorney (*Regime do Maior Acompanhado*, e.g., IC_321, daughter), were also expressed.

## 4 Discussion and conclusion

Most people with dementia are cared for by unpaid caregivers, especially in low-and middle-income countries (Prince et al., 2015), and in regions where a familialistic model of care prevails (del-Pino-Casado et al., 2011) as in Portugal. Cultural and political issues are influential on how the profile of caregivers and their needs varies across countries as well as on the burden they support (Meijer et al., 2022). Understanding the characteristics and needs of informal caregivers and PwD is a national priority reflected in the Portuguese Health Strategy for Dementia (Order n.° 5988/2018, 19th June) and the Informal Caregiver Statute (Law no.100/2019).

This research contributes to this knowledge by providing a comprehensive description of the sociodemographic, clinical, and psychosocial profiles of informal caregivers and PwD, along with insights into service utilization and unmet caregiver needs. The data is drawn from early adopters of iSupport-Portugal, collected upon their registration within the platform. This study differs from previous research in several important ways. First, in contrast to other national research (Movimento cuidar dos cuidadores informais, 2021), it focuses on specifically characterizing informal dementia caregivers, who are thought to be at higher risk of experiencing mental health problems than caregivers of people with other chronic conditions (WHO, 2015; Gilhooly et al., 2016). Second, it stands out for the number of variables collected to characterize the profile of dementia caregiving dyads, including a comprehensive array of sociodemographic, contextual, and psychosocial variables, both modifiable and non-modifiable. Other national studies have made an outstanding contribution in measuring psychosocial variables among informal dementia caregivers [e.g., Gonçalves-Pereira et al. (2019)]. Nevertheless, this study broadens the scope by measuring additional dimensions such as positive aspects of caregiving, caregivers’ use of community resources (e.g., psychoeducational groups), or the desire to institutionalize the PwD [a known predictor of actual institutionalization (Luppa et al., 2008)]. In addition, this research innovates in data collection methods by using a remote measurement tool (iSupport-Portugal) to collect nationwide data, thus overcoming the limitations of more circumscribed recruitment contexts (e.g., regional, or clinical recruitment).

Therefore, as a secondary by-product of the data collection through this platform, this research offers an opportunity to discuss the use of iSupport-Portugal as a RMT. Indeed, this study marks the first international exploration of iSupport beyond its original intervention purpose. From the preliminary measures taken to leverage iSupport-Portugal as a remote measurement tool (see Section 2.1, Preliminary Measures), several insights have been gained. Despite thorough preliminary testing of the platform (Teles et al., 2021) to prevent critical technical errors during data collection, the mobile version of iSupport-Portugal is still undergoing enhancements, impacting the convenience of participants’ self-completion of measurements. As illustrated in Tables 2–4, more than half of all eligible caregivers (*N* = 173) left at least one dimension unanswered when completing baseline measurements on iSupport’s platform. Preliminary testing of the data collection protocol indicated that completing the measurements took approximately 25 min, which was perceived as time-consuming but feasible. However, when measures are completed remotely and independently, without researcher prompting, caregivers may be more inclined to withdraw or fill in the measurements at different times or on separate days. Altogether, this suggests that improvements are needed regarding the accessibility and conciseness of data collection measures. Furthermore, enhancements are required for the automation of reminder systems to prompt platform users to complete assessment measures.

As for the main results of this study, iSupport-Portugal has attracted considerable attention from both informal caregivers and health/social care professionals. Within just 6 months, there were 365 full registrations, with 52.3% being by informal dementia caregivers. Consistent with national and international research [e.g., Wimo et al. (2018), Gonçalves-Pereira et al. (2019), Paúl et al., (2019), and ADI (2022)] caregivers registering on iSupport-Portugal were predominantly female and middle-aged. A higher representation of children (70.5%) was observed than in other Portuguese studies [e.g., 30.3% in Gonçalves-Pereira et al. (2019); 45.5% in Paúl et al. (2019)]. In line with trends seen in Mediterranean countries, a notable rate of cohabitation with the PwD (Barbosa and Matos, 2014) and the provision of high-intensity care (>20 h/week) (Hirst, 2005) were observed. In this sample, a high level of education is observed and there is a high representation of employed caregivers. Hence, this study distinguishes itself from previous research by examining caregivers’ needs in a context where achieving a work-life balance may be more challenging, and the choice to leave a career to provide full-time care may be less appealing (Flinn, 2018) or not be considered out of a necessity. Indeed, work-life balance concerns emerged in this study, as shown by the content analysis of text data (see section 3.2). Also pertinent to considerations on work-life balance is the provision of multigenerational care. As a relevant proportion of iSupport-Portugal users are children of PwD, this study gathered data on the number of offspring caregivers who live with their own children, of which over 70% were found to do so. While no significant association was found between being an offspring caregiver with cohabitating children and symptoms of burden, anxiety, or depression, previous studies have indicated that individuals who manage care responsibilities for both their parents and children - the so-called “sandwich” generation – have higher participation in the workforce and endure increased caregiving-related stress (Lei et al., 2023). Future research endeavors, with a larger user base of iSupport-Portugal, should delve deeper into this issue, given its substantial political implications.

The sociodemographic characteristics of PwD in this sample align with previous research, as does the clinical profile. Alzheimer’s disease is the most common subtype of dementia, consistent with most national epidemiological studies (Garcia et al., 1994; Nunes et al., 2010; Santana et al., 2015; Gonçalves-Pereira et al., 2017). Neuropsychiatric symptoms were reported in 94.1% of PwD, consistent with international research indicating a prevalence of 50 to 98% in community-dwelling PwD (Zhao et al., 2016). The severity scores were higher than those reported in a national study (Gonçalves-Pereira et al., 2019). Neuropsychiatric symptoms are increasingly recognized as core features of Alzheimer’s disease and other dementias, and a main contributor to caregiver psychological distress (Zhao et al., 2016) and institutionalization (Luppa et al., 2008). Therefore, the higher severity of neuropsychiatric symptoms in the care recipients of this sample may have prompted caregivers to seek online support. Apathy was the most common neuropsychiatric symptom, aligning with most research (Zhao et al., 2016). Appetite changes were more prevalent-than-usual (Zhao et al., 2016) in this study, but those may fall within the same subsyndrome category as apathy (Aalten et al., 2007).

Consistent with previous research, caregivers in this sample reported significant burden. However, for depression and anxiety symptoms, caregivers scored higher than in a recent Portuguese study that used the same measure (HADS M 6.5 and M 6.4 for anxiety and depression, respectively (Gonçalves-Pereira et al., 2019). This difference might be explained by the higher severity of neuropsychiatric symptoms reported in this sample. This is evidenced by the correlation of these symptoms with caregiver anxiety and depression, found in several other studies (Kim et al., 2021). The link between anxiety and employment status may also contribute to these elevated scores. Despite the high psychological distress observed in this sample, there was only a mild desire to institutionalize. The positive correlation found between caregiver burden and such desire highlights the need to intervene on modifiable factors to prevent the early placement of PwD.

This study has highlighted the low utilization of community support services by both PwD and their caregivers. Most of the caregiver support was coming from other informal sources. Less than a third of caregivers experiencing symptoms of depression and/or anxiety sought mental health support. The data does not indicate whether distressed caregivers not using mental health services were identified and referred by a health professional and chose not to use them or faced accessibility barriers. However, these findings may partly stem from underdiagnosis of depression and anxiety among informal dementia caregivers, as observed in other populations of caregivers (Zhang and Li, 2023). Recognizing that these caregivers are at higher risk of psychological distress, implementing routine screening in primary health settings, followed by referrals to mental health services and caregiver-centered interventions, is crucial to reduce the number of caregivers who are unsupported and untreated for their mental health concerns. Various factors contribute to the development of depression among dementia caregivers, including the characteristics and clinical profile of the PwD (Huang, 2022). Formulating individualized treatment plans and providing case management for both the PwD and their caregivers are crucial to address their needs effectively. Caregiver depression is increasingly impacting existing medical care, such as the utilization of emergency department services, underscoring the importance of addressing this issue from a healthcare cost management perspective as well. Upstream, early dementia diagnosis is crucial in helping caregivers adapt to their roles and access timely training and support interventions (de Vugt and Verhey, 2013).

Furthermore, caregivers supporting a PwD who was not utilizing community services tended to report more symptoms of anxiety and depression than those who were. This underscores the significance of enhancing the accessibility of community support services for PwD, including home care services, day, or night centers, cognitive or occupational therapy, and memory cafes, among others. In Portugal, specialized social responses for PwD are scarce, and the coverage rate for social responses catering to older individuals, including home care services and day centers, was only around 12% in 2021 (GEP - Gabinete de Estratégia e Planeamento, 2021).

Also, despite provisions in the Portuguese Informal Caregiver Statute that emphasize respite care as a fundamental aspect of caregiver support to mitigate burnout and promote physical and mental health, none of the caregivers in this sample used such services. The data do not allow for conclusions to be drawn about whether the caregivers in this sample needed or wanted respite care, and whether they encountered obstacles in obtaining it. However, from a regulatory perspective, to benefit from respite care within the scope of the Informal Caregiver Statute (Law No. 100/2019), individuals must have been formally recognized under this statute as a principal or non-principal caregiver. Since only principal caregivers are entitled to a monthly allowance, and since qualifying as such requires meeting conditions such as not having a job, caregivers who are employed may be unwilling to undergo the bureaucratic hurdle of applying for the statute. More than 60% of the caregivers in this study were employed and therefore would not qualify as primary caregivers. In addition, waiting lists for respite care are typically long, and family co-payments are often a barrier to accessing these services. All these barriers may impact the number of caregivers benefiting from the support measures outlined in the caregiver statute, including respite care (Instituto da Segurança Social IP, Administração Central do Sistema de Saúde I.P, 2021), necessitating additional political attention.

The findings from this research should be considered in light of its specific characteristics or limitations. In this study, several correlation coefficients indicating statistically significant associations demonstrated relatively low values. However, in behavioral sciences, correlation coefficients ranging from 0.3 to 0.5 are typically regarded as indicative of a moderate relationship, consistent with Cohen’s guidelines (Cohen, 1988). The relationships observed between the number of hours spent on caregiving and anxiety scores (*r*_s_ = 0.266), as well as between the positive aspects of caregiving and the severity of neuropsychiatric symptoms (*r*_s_ = −0.248), demonstrated weaker associations (*r* < 0.3) in this study.

As a potential limitation, the recruitment of caregivers for this research was not random and was conducted through the dissemination of iSupport-Portugal, which may have introduced volunteer bias. While this study may not guarantee national representativeness, convenience sampling aimed to avoid reproducing atypical situations. The dissemination efforts of iSupport-Portugal were extensive. Those involved collaboration with community projects and services, patient associations, communication with Portuguese Regional Health Administrations, and engagement with neurologists and psychiatrists in both private and public practice. Although the results may not generalize to the Portuguese population of informal dementia caregivers, the study sample is diverse and, overall, relatively typical in terms of sociodemographic characteristics, caregiving context, and caregivers’ psychological needs. This includes the high rate of women, caregivers in cohabitation with the PwD, and high psychological distress. Nevertheless, caregivers who are children of the PwD, employed, and highly educated may be overrepresented in this sample. This fact is possibly due to the use of digital means for data collection and the program’s appeal to newer generations of caregivers. A recent Portuguese study reported a higher-than-usual percentage of highly educated caregivers (Gonçalves-Pereira et al., 2019), although the prevailing description has been of lower education levels. Moreover, participants in caregiving studies are often recruited through community projects that are less accessible to employed, younger, and more educated caregivers, making this study potentially more inclusive in reaching caregivers who are typically underrepresented. As political investments across Europe to close the digital divide begin to yield results and digital natives assume caregiver roles, iSupport-Portugal may be able to reach a more diverse group of caregivers.

In conclusion, the baseline data from early adopters of iSupport-Portugal suggest significant psychological distress and unmet practical, emotional, and informational needs among informal caregivers of PwD. Despite limited utilization of community support services by both caregivers and PwD, caregivers reported higher levels of anxiety and depression when these services were not used. The ability to meet the needs of a growing number of PwD and ensure they can continue to receive quality care at home hinges on multiple factors, including the well-being of informal caregivers. Identifying caregiver needs in a timely manner and providing proactive interventions is therefore essential. iSupport-Portugal can serve as a valuable remote tool for collecting data and informing on the profile, needs, and resources of dementia care dyads. In the short term, this information could lay the groundwork for expanding and enhancing the training and support offered through iSupport-Portugal. This could involve creating new training modules or improving existing ones. Moreover, caregiver profiles could inform a recommendation algorithm within iSupport, directing users to modules and lessons tailored to their specific needs. On a broader scale, the insights gleaned from this study may inform both practice and policy. They underscore the underutilization of support services for caregivers, an area targeted for improvement politically both nationally, as evidenced in the recent Informal Caregiver Statute (Law no.100/2019), and internationally (WHO, 2017).

Upcoming research endeavors with iSupport-Portugal will focus on following up a cohort of caregivers on the desire to institutionalize and the actual institutionalization of PwD. This should allow to examine whether the sociodemographic and psychosocial variables collected at baseline and described in this study can be used to predict these outcomes.

## Data availability statement

The raw data supporting the conclusions of this article will be made available by the authors, without undue reservation.

## Ethics statement

The studies involving humans were approved by the Ethics Committee for Health of the Faculty of Medicine of the University of Porto (ref: 76/CEFMUP/2022). The studies were conducted in accordance with the local legislation and institutional requirements. The participants provided their written informed consent to participate in this study.

## Author contributions

ST: Conceptualization, Data curation, Formal analysis, Funding acquisition, Investigation, Methodology, Project administration, Writing – original draft, Writing – review & editing. SA: Methodology, Writing – review & editing. OR: Methodology, Writing – review & editing. AlF: Methodology, Writing – review & editing. AnF: Methodology, Writing – review & editing. CP: Conceptualization, Funding acquisition, Methodology, Project administration, Writing – review & editing.
